# Abwarten oder Radiochirurgie bei asymptomatischen Vestibularisschwannomen

**DOI:** 10.1007/s00066-023-02142-1

**Published:** 2023-08-21

**Authors:** Olaf Wittenstein, Jürgen Dunst

**Affiliations:** Klinik für Strahlentherapie/Radioonkologie, UKSH, Campus Kiel, Kiel, Deutschland

## Hintergrund

Asymptomatische Vestibularisschwannome werden meistens zufällig bei MRT-Untersuchungen des Hirns entdeckt. Diese meistens kleinen Tumoren wachsen langsam (im Durchschnitt etwa 1 mm pro Jahr), und die Patienten bleiben oft jahrelang vollkommen beschwerdefrei. Jede Intervention muss sich deshalb an diesem günstigen Spontanverlauf messen. Für asymptomatische kleine Tumoren wird deshalb als sinnvolle Option lediglich eine Überwachung mit regelmäßigen MRT-Kontrollen empfohlen, die sog. Wait-and-scan-Strategie [1]. In einer norwegischen Studie wurde jetzt gezeigt, dass eine Radiochirurgie zu einer signifikanten Volumenreduktion führt ohne funktionelle Nachteile [2].

## Patienten und Methodik

Die Studie wurde am Haukeland-Universitätskrankenhaus in Bergen durchgeführt, dem nationalen Behandlungszentrum für Vestibularisschwannome (VS) in Norwegen. Patienten mit neu diagnostizierten VS mit einem Durchmesser von maximal 2 cm wurden randomisiert und erhielten entweder eine Radiochirurgie (Leksell Gamma Knife, 1 × 12 Gy, dosiert auf die 40 %- bis 60 %-Isodosis am Tumorrand) oder keine Therapie. Beide Gruppen wurden anschließend weiter beobachtet und erhielten einmal jährlich eine kontrastmittelverstärkte MRT; primärer Endpunkt war die Größenänderung des Tumors nach 4 Jahren. Bei den jährlichen Untersuchungen wurden außerdem klinische Untersuchungen, audiometrische und vestibuläre Tests und Befragungen der Patienten zum patientenorientierten Outcome durchgeführt; insgesamt wurden 26 sekundäre Endpunkte analysiert.

## Ergebnisse

Von Oktober 2014 bis Oktober 2017 wurden 100 Patienten randomisiert; 98 wurden ausgewertet. Das mediane Alter war 54 Jahre; 42 % waren Frauen. Nach Radiochirurgie wurden 94 % der Patienten nicht weiter behandelt, die übrigen 6 % wurden wegen Rezidiven operiert (4 %) oder mit erneuter Radiochirurgie (2 %) behandelt. In der zunächst unbehandelten Gruppe war eine Therapie innerhalb von vier Jahren bei 56 % der Patienten nicht nötig, 42 % erhielten eine Radiochirurgie und 2 % eine Operation wegen Tumorwachstum. Bezüglich des primären Endpunkts ergab sich ein signifikanter Vorteil in der Radiochirurgiegruppe mit einer Volumenabnahme von 13 %, während das Tumorvolumen in der zunächst nicht behandelten Gruppe nach 4 Jahren um 51 % angestiegen war. In 25 der 26 sekundären Endpunkte gab es keine signifikanten Unterschiede. RT-assoziierte Komplikationen wurden nicht beobachtet.

## Bewertung der Autoren

In dieser Studie konnte erstmals klar gezeigt werden, dass eine frühe Radiochirurgie einen signifikanten positiven Effekt auf das Größenwachstum von asymptomatischen VS hat und dass damit keine funktionellen Nachteile verbunden sind. Die Autoren halten die Ergebnisse für wichtig, um Patienten optimal beraten zu können. Für eine detailliertere Bewertung zum optimalen Vorgehen sind längere Nachbeobachtungszeiten erforderlich.

## Kommentar

Bei zufällig entdeckten asymptomatischen Vestibularisschwannomen muss eine Übertherapie vermieden werden. Mehrere nichtrandomisierte Studien (zusammen aber die beste verfügbare Evidenz) zeigen eindeutige Vorteile einer Radiochirurgie gegenüber einer Operation im funktionellen Outcome [3–7]. Deshalb kommt für diese asymptomatischen Tumoren, wenn überhaupt, nur eine Radiochirurgie als Alternative zu einem lediglich abwartenden Vorgehen infrage. Diese norwegische Studie zeigt erstmals, dass eine frühe Radiochirurgie gegenüber dem günstigen Spontanverlauf keine funktionellen Nachteile hat, aber eine Größenzunahme verhindert. Die Studie zeigt auch, dass Abwarten und Aufschieben der Behandlung bis zur relevanten Größenprogression ein adäquates Konzept ist. Eine eindeutige Überlegenheit des einen oder anderen Vorgehens besteht anhand dieser Daten nicht, zumindest nicht über den für diese Krankheit relativ kurzen Beobachtungszeitraum von vier Jahren. Das ist also eine typische Situation für ein „shared decision-making“. Deshalb können und sollten wir anbieten, dass diese Patienten eine entsprechende Beratung und die Verlaufskontrollen in einem Radiochirurgiezentrum erhalten. Die Datenlage bzgl. der Länge der Kontrollintervalle (in der Studie wurden - wie in den EANO Leitlinien empfohlen - jährliche MRT-Untersuchungen durchgeführt) ist jedoch dünn. Hinzu kommt das Risiko der Pseudoprogression, dass in den ersten Jahren nach Radiochirurgie für Verunsicherung sorgen kann. Vielleicht werden uns diesbezüglich die 10-Jahres-Follow-Up-Daten dieser Norwegischen Studie (zu erwarten ca. 2027) weitere Informationen liefern.

*Olaf Wittenstein und Jürgen Dunst, Kiel*
